# Predictors of BMI reduction with phentermine/topiramate in adolescents with obesity

**DOI:** 10.1038/s41366-025-01821-6

**Published:** 2025-06-15

**Authors:** Megan O. Bensignor, Rebecca L. Freese, Kyle D. Rudser, Aaron S. Kelly, Alicia Kunin-Batson, Amy C. Gross, Carolyn Bramante, Winnie Shih, Craig Peterson, Claudia K. Fox

**Affiliations:** 1 Department of Pediatrics, Medical School, University of Minnesota, Minneapolis, MN USA; 2 Center for Pediatric Obesity Medicine, University of Minnesota Medical School, Minneapolis, MN USA; 3 Clinical and Translational Science Institute, Biostatistical Design and Analysis Center, University of Minnesota, Minneapolis, MN USA; 4 Division of Biostatistics and Health Data Science, School of Public Health, University of Minnesota, Minneapolis, MN USA; 5 Department of Medicine, Medical School, University of Minnesota, Minneapolis, MN USA; 6 VIVUS LLC, Campbell, CA USA

**Keywords:** Paediatrics, Translational research

## Abstract

**Background/Objectives:**

Obesity treatment can produce variable outcomes for different individuals. The aim of this analysis in adolescents with obesity was to investigate if baseline participant characteristics associated with BMI reduction from baseline to 56 weeks when treated with mid- or top-dose phentermine/topiramate (PHEN/TPM) compared to placebo.

**Methods:**

A secondary analysis of a randomized, double-blind, placebo-controlled, clinical trial evaluating PHEN/TPM in adolescents with obesity was conducted. Participants, aged 12 to <17 years with a BMI ≥95th percentile, were randomly assigned in a 1:1:2 ratio to receive either placebo, mid-dose (PHEN/TPM 7.5 mg/46 mg) or top-dose (PHEN/TPM 15 mg/92 mg). Baseline characteristics included in the analysis were BMI, age, sex, race/ethnicity, pubertal status, diabetes status, depression status, cognitive function score, and quality of life score. The primary analysis used linear regression with BMI percent change from baseline to 56 weeks as the outcome with either mid- or top-dose PHEN/TPM compared to placebo.

**Results:**

Two-hundred twenty-two participants were included in the final analysis. None of the baseline characteristics were statistically significantly associated with BMI reduction with mid- or top-dose PHEN/TPM compared to placebo.

**Conclusions:**

Baseline characteristics were not predictive of BMI reduction with either dose of PHEN/TPM compared to placebo in adolescents with obesity.

## Introduction

Obesity, which currently affects about 21% of children and adolescents in the United States, is a multifactorial chronic disease with variable treatment response [1, 2]. Treatment options, including lifestyle and behavioral modification, pharmacotherapy, and bariatric surgery, may result in clinically significant BMI reduction for some adolescents while others may experience no change or increased BMI [3–6]. A personalized approach is important as there are now four United States Food and Drug Administration (FDA) approved medications for the long-term treatment of adolescent obesity: semaglutide 2.4 mg weekly [7], liraglutide 3.0 mg daily [8], orlistat [9], and phentermine/topiramate (PHEN/TPM) [10].

The randomized, placebo-controlled trial of PHEN/TPM for the treatment of adolescent obesity, enrolled participants aged 12 to <17 years with obesity [11]. The results of this study describe a heterogeneous response as previously described with other obesity treatments [3–5]. At 56 weeks, 38.9% of participants receiving mid-dose and 46.9% receiving top dose PHEN/TPM compared to 5.4% receiving placebo, had a ≥5% BMI reduction [11]. To date, predictors of BMI reduction with PHEN/TPM in a pediatric population have not been investigated.

We conducted a secondary analysis examining predictors of BMI reduction response with PHEN/TPM compared to placebo using data from the adolescent PHEN/TPM clinical trial [11]. The primary objective was to identify baseline participant characteristics predictive of BMI reduction with mid-and top-dose PHEN/TPM treatment to aid in clinical decision-making for adolescents with obesity.

## Subjects and methods

### Overview

This analysis was conducted using data collected from “A Phase IV Safety and Efficacy Study of VI-0521 in Adolescents with Obesity” (Clinicaltrials.gov # NCT03922945) [11]. This randomized, double-blind, placebo-controlled trial was designed to evaluate the safety and efficacy of PHEN/TPM in adolescents with obesity over 56 weeks. Participants were randomly assigned in a 1:1:2 ratio to receive either placebo, mid-dose (7.5 mg/46 mg) or top-dose (15 mg/92 mg) PHEN/TPM. The trial protocol and primary outcomes have been previous published [11]. Eligible participants were 12 to <17 years of age with BMI ≥95th percentile for age and sex, and had a Tanner (pubertal) stage >1, history of insufficient weight reduction or maintenance with a lifestyle modification program. The full list of inclusion and exclusion criteria is provided in the Supplementary Appendix of the original publication [11].

### Measures

#### Demographics

Sex, age, and race and ethnicity were collected at the baseline visit. Due to limited numbers of participants, race and ethnicity were combined and collapsed into two race/ethnicity categories: (1) participants who identified as both white race and non-Hispanic ethnicity (“non-Hispanic white”), and (2) participants who identified as Hispanic ethnicity and/or Black/African American, Asian, Native American or Alaskan Native, Native Hawaiian or Other Pacific Islander, Asian or other (“Hispanic and/or not white”).

#### Anthropometrics

Baseline and week 56 height and weight were used to calculate BMI.

#### Tanner Staging

Tanner Staging, a sex-specific five-step rating of secondary sexual characteristics, was measured to assess pubertal maturation. Pubertal stage was categorized as “early” (Tanner 2 and 3) and “late” (Tanner 4 and 5).

#### Glycemic status

An oral glucose tolerance test (OGTT) obtained at baseline determined a participant’s baseline glycemic status. The OGTT used a 75 g oral glucose load; blood samples were obtained at fasting and at 2 h post glucose load for evaluation of both glucose and insulin levels. Participants with a fasting glucose level between 100–125 mg/dL or 2-h glucose level between 140–199 mg/dL were classified as “prediabetes” [12]. Participants with a fasting glucose level ≤100 mg/dL or 2-h glucose level ≤139 mg/dL were classified as “normoglycemia.”

#### The Patient Health Questionnaire-9 Modified for Teens (PHQ-9)

The PHQ-9 is a 9-item, self-administered instrument to screen for and assess the severity of depression in adolescents. Depression severity was determined according to PHQ-9 guidelines: none = 0 to 4; mild = 5–9; moderate = 10–14; moderately severe = 15–19; severe = 20–27 [13]. Participants who screened with PHQ-9 ≥ 10 or more were excluded from the study. Depression as a predictor was categorized into “none (0–4)” and “mild (5–9).”

#### Cambridge Neuropsychological Test Automated Battery (CANTAB)

Baseline cognitive function was assessed using spatial span test from the CANTAB. The spatial span test was used to assess visuospatial working memory capacity, which is one domain of executive function [14]. The raw score describes the longest sequence successfully recalled, with sequences ranging from two to nine and a higher score indicating better performance [14]. CANTAB as a predictor was categorized into high (≥7) and low (<7).

#### The Impact of Weight on Quality of Life-Kids (IWQOL-Kids)

The IWQOL-Kids is a 27-item, self-administered questionnaire validated for use in adolescents aged 11–19 years old [15]. This questionnaire was designed to evaluate the impact of excess weight on quality of life domains including Physical Comfort, Body Esteem), Social Life, and Family Relations [15]. The IWQOL-Kids results in transformed scores that range from 0–100, with higher scores indicating better quality of life. IWQOL as a predictor was categorized as high (≥85) and low (<85).

### Statistical analysis

Only baseline data were used for this analysis. Descriptive analyses of baseline characteristics and measures included means with standard deviations for continuous variables and counts with frequencies for categorical variables. Linear interpolation was used to estimate BMI at the 56 week target date for participants whose BMI was collected more than 30 days past the target date (*n* = 10) using the late measured BMI and the closest measured BMI prior to the week 56 target visit. Multiple imputation was used for records missing 56 week BMI measures via 200 imputations based on the randomization group and the randomization stratification factors (age group and sex). The primary analysis used linear regression with BMI percent change from baseline to 56 weeks as a continuous outcome. Predictor variables of age (12–14 vs. 15–16), sex (male vs. female), race and ethnicity (non-Hispanic and white vs. Hispanic and/or not white), baseline BMI category (between 95–99th age- and sex-specific percentile vs. ≥99th), Tanner stage (4 and 5 vs. 2 and 3), glycemic status (normoglycemic vs. prediabetes), depression status (no depression vs. mild depression), CANTAB spatial span score (high (≥7) vs. low (<7)), and IWQOL scores (high (≥85) vs. low (<85)) were each evaluated in a separate model using an interaction with the treatment group (placebo, mid-dose PHEN/TPM and top-dose PHEN/TPM). All models adjusted for baseline value of BMI and randomization stratification factors of age group and sex, and used robust variance estimation for confidence intervals and *p* values. For each predictor, results were pooled across imputed datasets. All *p* values are two-sided and considered at the 0.05 level for statistical significance. Analyses were performed using R (v4.2.3; R Core Team 2023) (25) and the mice package (26).

## Results

A total of 222 participants were included in this analysis: 56 in the placebo group, 54 in the mid-dose group, and 112 in the top-dose group. Participant characteristics described by separate racial and ethnic categories and as a combined race/ethnicity category for the analysis are outlined in Table 1.

**Table 1:** Baseline characteristics of participants.

	Placebo	Mid-dose PHEN/TPM	Top-dose PHEN/TPM
*N*	56	54	112
Age, years (Mean ± SD)	14.0 (1.4)	14.1 (1.3)	14.0 (1.3)
Age category, *N* (%)^a^			
12–14 years	34 (60.7)	33 (61.1)	68 (60.7)
15–16 years	22 (39.3)	21 (38.9)	44 (39.3)
Sex, *N* (%)			
Female	30 (53.6)	28 (51.9)	63 (56.2)
Male	26 (46.4)	26 (48.1)	49 (43.8)
Race, *N* (%)			
American Indian or Alaska Native	0 (0.0)	0 (0.0)	1 (0.9)
Asian	0 (0.0)	0 (0.0)	1 (0.9)
Black or African American	10 (17.9)	14 (25.9)	36 (32.1)
Other^b^	4 (7.1)	4 (7.4)	4 (3.6)
White	42 (75.0)	36 (66.7)	70 (62.5)
Ethnicity, *N* (%)			
Hispanic or Latino	13 (23.6)	25 (47.2)	33 (29.5)
Non-Hispanic or Non-Latino	42 (76.4)	28 (52.8)	79 (70.5)
Missing	1	1	0
Race/Ethnicity, *N* (%)			
Hispanic and/or not White	27 (48.2)	38 (71.7)	72 (64.3)
Non-Hispanic White	29 (51.8)	15 (28.3)	40 (35.7)
Missing	0	1	0
Baseline BMI, kg/m^2^ (Mean ± SD)	36.4 (6.4)	36.9 (6.7)	39.0 (7.5)
% of the 95th BMI percentile (Mean ± SD)	136.7 (23.0)	138.7 (25.2)	147.0 (28.7)
Tanner stage, *N* (%)			
Stages 2 and 3	23 (41.1)	16 (29.6)	37 (33.0)
Stages 4 and 5	33 (58.9)	38 (70.4)	75 (67.0)
Glycemic status, *N* (%)			
Normoglycemic	40 (75.5)	37 (69.8)	82 (77.4)
Prediabetes	13 (24.5)	16 (30.2)	24 (22.6)
Missing	3	1	6
Depression status, *N* (%)			
None	50 (89.3)	45 (83.3)	83 (76.1)
Mild	6 (10.7)	9 (16.7)	26 (23.9)
Missing	0	0	3
CANTAB-Spatial Span Score (Mean ± SD)	6.6 (1.4)	6.8 (1.5)	6.8 (1.4)
CANTAB-Spatial Span Score, *N* (%)			
Low (<7)	27 (48.2)	17 (31.5)	52 (46.4)
High (≥7)	29 (51.8)	37 (68.5)	60 (53.6)
IWQOL-Kids total score (Mean ± SD) [Missing]	87.6 (10.3)	85.5 (12.9)	82.6 (14.3) [2]
IWQOL-Kids total score, *N* (%)			
Low (<85)	18 (32.1)	25 (46.3)	48 (43.6)
High (≥85)	38 (67.9)	29 (53.7)	62 (56.4)
Missing	0	0	2

*BMI* body mass index, *CANTAB* Cambridge Neuropsychological Test Automated Battery, *IWQOL* The Impact of Weight on Quality of Life.

^a^Age category was the randomization stratification variable and adjusted for in models.

^b^Other races included: Mexican, Middle Eastern, and Multiple.

**Fig. 1:**
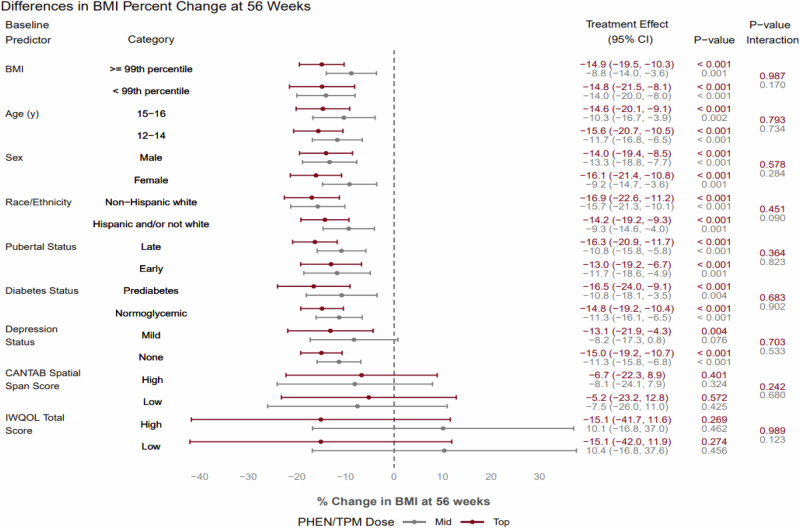
Percentage change in BMI from baseline to 56 weeks by PHEN/TPM dose and baseline characteristic. Difference in percent change in BMI from baseline to 56 weeks by PHEN/TPM dose relative to placebo (treatment effect) by predictor subgroups. The interaction *p* value corresponds to a test of different treatment effect between predictor subgroups for a given PHEN/TPM dose.

None of the baseline characteristics had a statistically-significant treatment difference for continuous BMI reduction with PHEN/TPM (at either mid- or top-dose) compared to placebo (Fig. 1).

## Discussion

The results of this secondary analysis of the clinical trial evaluating the effect of PHEN/TPM in adolescents with obesity highlight that both doses of PHEN/TPM are effective for BMI reduction regardless of age, sex, race and ethnicity, pubertal stage, baseline BMI, depression, glycemic status, executive functioning, and quality of life.

Predictors of pediatric response to phentermine/ topiramate combination therapy are under-studied. Although retrospective chart reviews from a pediatric weight management clinic have shown that alanine aminotransferase elevations and higher food responsiveness may predict less BMI reduction with phentermine monotherapy [16], baseline patient characteristics have not been associated with greater BMI reduction with topiramate monotherapy [17]. In adults, topiramate when used for epilepsy resulted in greater weight reduction for patients with higher BMI [18, 19], younger age [19], and female sex [20]. These results differ from our secondary analysis possibly due the previous studies being observational [19] or a secondary analysis of anticonvulsant trial [18] and with varying topiramate doses [18–20]. The main limitation of this secondary analysis is that the trial was not originally designed to detect response differences by demographics or clinical variables.

In conclusion, no baseline characteristics were associated with greater BMI reduction with PHEN/TPM in adolescents with obesity, suggesting that this medication at either dose may result in similar BMI reduction regardless of age, sex, glycemic status, or baseline BMI.

## Data Availability

The data that support the findings of this study are available from VIVUS LLC but restrictions apply to the availability of these data, which were used under license for the current study, and so are not publicly available. Data are however available from the authors upon reasonable request and with permission of VIVUS LLC.

## References

[1] Hampl SE, Hassink SE, Skinner AC, Armstrong SC, Barlow SE, Bolling CF, et al. Clinical practice guideline for the evaluation and treatment of children and adolescents with obesity. Pediatrics. 2023;151:e2022060640. 10.1542/peds.2022-060640. 10.1542/peds.2022-060640 36622115

[2] Emmerich SD, Fryar CD, Stierman B, Gu Q, Afful J, Ogden CL. Trends in obesity-related measures among US children, adolescents, and adults. JAMA. 2025:e2427676. 10.1001/jama.2024.27676. 10.1001/jama.2024.27676 PMC11826431 39946125

[3] Kelly AS, Auerbach P, Barrientos-Perez M, Gies I, Hale PM, Marcus C, et al. A randomized, controlled trial of liraglutide for adolescents with obesity. N Engl J Med. 2020;382:2117–28. 32233338 10.1056/NEJMoa1916038

[4] Danielsson P, Kowalski J, Ekblom Ö, Marcus C. Response of severely obese children and adolescents to behavioral treatment. Arch Pediatr Adolesc Med. 2012;166:1103–8. 23108856 10.1001/2013.jamapediatrics.319

[5] Johnston CA, Tyler C, Palcic JL, Stansberry SA, Gallagher MR, Foreyt JP. Smaller weight changes in standardized body mass index in response to treatment as weight classification increases. J Pediatr. 2011;158:624–7. 21035822 10.1016/j.jpeds.2010.09.049

[6] Ryder JR, Kaizer AM, Jenkins TM, Kelly AS, Inge TH, Shaibi GQ. Heterogeneity in response to treatment of adolescents with severe obesity: the need for precision obesity medicine. Obesity. 2019;27:288–94. 30677258 10.1002/oby.22369 PMC6352902

[7] Novo Nordisk. FDA approves once-weekly Wegovy® injection for the treatment of obesity in teens aged 12 years and older. 2022. https://www.novonordisk-us.com/media/news-archive/news-details.html?id=151389.

[8] Bacha F. FDA approval of GLP-1 receptor agonist (liraglutide) for use in children. Lancet Child Adolesc Health. 2019;3:595–7. 10.1016/S2352-4642(19)30236-6. 31345736 10.1016/S2352-4642(19)30236-6

[9] U.S. Food and Drug Administration. Orlistat (marketed as Allli and Xenical) information. 2015. https://www.fda.gov/drugs/postmarket-drug-safetyinformation-patients-and-providers/orlistat-marketed-alli-and-xenical information#:~:text=Xenical%20(orlistat%20120mg)%20was%20approved,weight%20after%20prior%20weight%20loss.

[10] U.S. Food and Drug Administration. Highlights of prescribing information: Qsymia (phentermine and topiramate extended-release). 2013. https://www.accessdata.fda.gov/drugsatfda_docs/nda/2012/022580Orig1s000LBL.pdf.

[11] Kelly AS, Bensignor MO, Hsia DS, Shoemaker AH, Shih W, Peterson C, et al. Phentermine/topiramate for the treatment of adolescent obesity. NEJM Evid. 2022;1:1–11. 10.1056/evidoa2200014 PMC10035585 36968652

[12] American Diabetes Association Professional Practice Committee. 2. Diagnosis and classification of diabetes: standards of care in diabetes-2024. Diabetes Care. 2024;47:S20–42. 10.2337/dc24-S002. 38078589 10.2337/dc24-S002 PMC10725812

[13] Nandakumar AL, Vande Voort JL, Nakonezny PA, Orth SS, Romanowicz M, Sonmez AI, et al. Psychometric properties of the Patient Health Questionnaire-9 Modified for major depressive disorder in adolescents. J Child Adolesc Psychopharmacol. 2019;29:34–40. 30388048 10.1089/cap.2018.0112 PMC6354604

[14] Teixeira RAA, Zachi EC, Roque DT, Taub A, Ventura DF. Memory span measured by the spatial span tests of the Cambridge Neuropsychological Test Automated Battery in a group of Brazilian children and adolescents. Dement Neuropsychol. 2011;5:129–34. 29213735 10.1590/S1980-57642011DN05020012 PMC5619309

[15] Kolotkin RL, Zeller M, Modi AC, Samsa GP, Quinlan NP, Yanovski JA, et al. Assessing weight-related quality of life in adolescents. Obesity. 2006;14:448–57. 16648616 10.1038/oby.2006.59 PMC2374918

[16] Bomberg EM, Clark J, Rudser KD, Gross AC, Kelly AS, Fox CK. Effectiveness and predictors of weight loss response to phentermine plus lifestyle modifications among youth in a paediatric weight management clinical setting. Pediatr Obes. 2024;19:e13143. 38886982 10.1111/ijpo.13143 PMC11239309

[17] Bomberg EM, Clark J, Rudser KD, Gross AC, Kelly AS, Fox CK. Clinical effectiveness and predictors of response to topiramate plus lifestyle modification in youth with obesity seen in a weight management clinical setting. Front Endocrinol. 2024;15:1369270. 10.3389/fendo.2024.1369270 PMC11116594 38800488

[18] Ben-Menachem E, Axelsen M, Johanson EH, Stagge A, Smith U. Predictors of weight loss in adults with topiramate-treated epilepsy. Obes Res. 2003;11:556–62. 12690085 10.1038/oby.2003.78

[19] El Yaman SH, Mroueh SM, Sinno DD, Mikati MA. Long-term patterns of weight changes during topiramate therapy: an observational study. Neurology. 2007;69:310–1. 17636070 10.1212/01.wnl.0000265853.66458.82

[20] Klein KM, Theisen F, Knake S, Oertel WH, Hebebrand J, Rosenow F, et al. Topiramate, nutrition and weight change: a prospective study. J Neurol Neurosurg Psychiatry. 2008;79:590–3. 18077476 10.1136/jnnp.2007.136929

